# Efficacy and safety of pregabalin in the management of low back pain: a comprehensive meta-analysis

**DOI:** 10.3389/fphar.2025.1659531

**Published:** 2025-09-08

**Authors:** Carlos Cordero‐García, Judith Sánchez‐Raya, Tamara L. Rodríguez‐Araya, María Dolores López‐Alarcón, Eva Trillo‐Calvo, Jerónimo Balsalobre‐Aznar, Maria Pérez‐Páramo

**Affiliations:** ^1^ Department of Physical Medicine and Rehabilitation, Hospital Universitario Juan Ramón Jiménez, Huelva, Spain; ^2^ Physical Medicine and Rehabilitation Department, Hospital Universitari Vall d'Hebron, Barcelona, Spain; ^3^ Rheumatology Service, Hospital Clinic, Barcelona, Spain; ^4^ Pain Unit, General University Hospital of Valencia, Valencia, Spain; ^5^ Primary Care, Aragon Institute for Health Research (IIS Aragón), Primary Care Center Campo de Belchite, Zaragoza, Spain; ^6^ Rheumatology Service, Hospiten, Santa Cruz de Tenerife, Spain; ^7^ Medical Department, Viatris, Madrid, Spain

**Keywords:** low back pain, radiculopathy, sciatica, neuropathic pain, meta-analysis

## Abstract

**Introduction:**

Low back pain (LBP) is one of the most prevalent chronic pain conditions that affects nearly 50% of the population. Its complex pathophysiology may involve both nociceptive and neuropathic mechanisms and is often resistant to standard treatment. Pregabalin has emerged as a potential alternative owing to its mechanism of action, the inhibition of excitatory neurotransmitter release. This meta-analysis aimed to evaluate the efficacy and safety of pregabalin in managing LBP.

**Methods:**

A systematic review of three major databases was conducted following the PRISMA guidelines. Studies were included if they were comparative studies of pregabalin with placebo or other pain medications, focusing on adult patients with LBP. Data were extracted on efficacy outcomes including pain reduction, anxiety, depression, quality of life, quality of sleep, disability, and adverse events. Statistical analysis was performed using Review Manager 5.4.1.

**Results:**

A total of 18 studies (*n =* 5,000) were included. Pregabalin demonstrated significant pain reduction at 4 weeks (Standardized Mean Difference (SMD) = −0.64, 95% Confidence Interval (CI) = −1.09 to −0.20), 6 weeks (SMD = −0.72, 95% CI = −1.15 to −0.29), and 8 weeks (SMD = −0.50, 95% CI = −0.71 to −0.29) compared to control group. Pregabalin also showed a significant greater improvement in anxiety (Mean Difference (MD) = −1.38, 95% CI = −1.74 to −1.02, p < 0.00001), depression (MD = −1.40, 95% CI = −1.71 to −1.08, p < 0.00001), quality of life (SMD = 0.22, 95% CI = 0.07 to 0.37, p = 0.003) and sleep quality (SMD = −0.61, 95% CI = −0.87 to −0.36, p < 0.00001). There were no significant differences regarding disability and adverse events.

**Conclusion:**

Pregabalin in the treatment of neuropathic LBP demonstrated significant improvements in pain relief, associated symptoms of anxiety and depression, and enhancements in quality of life and sleep quality. In addition, it exhibits a favorable safety profile. Nevertheless, these findings should be interpreted with caution due to the limited quality of the evidence and the inadequate reporting of pain etiology in several included studies.

## 1 Introduction

Low back pain (LBP) is one of the most prevalent pain conditions and a leading cause of disability worldwide. Its complex pathophysiology may involve both nociceptive and neuropathic mechanisms, contributing to the heterogeneous nature of the condition. In 2020, LBP affected 619 million (95% uncertainty interval 554–694) people globally (GBD, 2021 Low Back Pain Collaborators, 2023).

In LBP, nociceptive pain results from activation of nociceptors that innervate ligaments, joints, muscles, fascia and tendons as a response to tissue injury or inflammation and biomechanical stress. Neuropathic back pain describes pain arising from injury or disease directly affecting the nerve roots that innervate the spine and lower limbs, and pathological invasive innervation of the damaged lumbar discs. Chronic LBP is increasingly considered to be a mixed pain syndrome consisting of both nociceptive and neuropathic components, and it has been suggested that neuropathic components in chronic LBP may be under-recognized and therefore undertreated (Baron et al., 2016).

This mixed pain condition is notoriously difficult to manage and resistant to treatments (Ghazisaeidi et al., 2023; Sharma and McAuley, 2022; Mák et al., 2021). Pain is also associated with a significant decline in quality of life and leads to both direct and indirect costs (Ghazisaeidi et al., 2023; Sharma and McAuley, 2022; Mák et al., 2021; Shetty et al., 2022). Studies suggest that nearly a quarter of individuals with LBP suffer from psychiatric comorbidities, such as depression and anxiety, which has led to advocate for psychiatric screenings in these patients (Dickson et al., 2023; Hu et al., 2022). Furthermore, the indirect costs of managing lumbar pain can reach up to 68% of total costs (Morera-Domínguez et al., 2010). Pharmacy costs alone may account for as much as 13% of the total expenses related to this condition while the cost of physical therapy account for up to 17% (Dagenais et al., 2008). Additionally, all the costs incurred, such as those resulting from reduced productivity at work, are significant, because LBP is the leading cause of disability in the United States and the main reason for absenteeism (Areias et al., 2023).

Current treatments for lower back pain, such as NSAIDs and opioids, often have significant drawbacks. NSAIDs, for instance, are associated with considerable health risks, including gastrointestinal toxicity, which can impose a substantial economic burden (Kikuchi et al., 2021). The majority of guidelines for the treatment of low back pain recommend paracetamol as the first-line option, with NSAIDs (that are readily accessible in various types and brands, both over-the-counter and by prescription) as a subsequent choice if paracetamol proves inadequate. While most NSAIDs are taken in oral preparations, topical formulations are also available to avoid systemic side effects, particularly in patients with allergies to specific medications, gastroesophageal reflux disease, and cardiac disease (Enthoven et al., 2016; Wang and Doan, 2024).

In addition, a Cochrane review noted by Cashin et al. (2023) found that opioids can lead to adverse events like nausea, headaches, constipation, and dizziness, which can severely affect a patient’s quality of life. The rapid escalation of the opioid epidemic reveals critical issues like addiction and dependency, complicating long-term treatment and management. This situation underscores the urgent need for alternative strategies to address these challenges effectively (Damiescu et al., 2021).

Although recent national guidelines have offered conflicting recommendations on the role of opioids in treating chronic LBP, opioids remain widely used in both Europe and North America. The prolonged use of opioids for chronic non-cancer pain has played a significant part in the opioid crisis in North America, contributing to a rise in opioid prescriptions, non-medical use, and the associated increase in mortality rates (Petzke et al., 2020). The guidelines from the European Medicines Agency and the American CDC highlight the need to assess physical dependence, abuse, and addiction in opioid trials, but these outcomes were not analyzed in the most systematic reviews for chronic LBP (Petzke et al., 2020).

For all these reasons, the need arose to seek more treatments that could be incorporated into clinical guidelines. Pregabalin emerges as treatment for neuropathic lumbar pain due to its ability to selectively bind to the α2-δ subunit of voltage-dependent calcium channels, inhibiting calcium influx, reducing excitatory neurotransmitter release and, as a consequence, reducing nerve impulse and pain sense (Taylor et al., 2007). This molecular action decreases pain signal transmission, alleviates symptoms, and enhances functionality, thus highlighting its potential in the management of LBP (Taylor et al., 2007).

Original studies on the pharmacological management of low back pain revealed mixed effectiveness across treatments. Robertson et al. (2019) favour gabapentin (GBP) for its minimal adverse effects and significant pain relief, whereas Sakai et al. (2015) and Mathieson et al. (2017) found no substantial benefits of pregabalin over opioids or placebo, respectively. Park et al. (2024) suggest that the choice between pregabalin and limaprost should be tailored to the individual, while Kalita et al. (2014) highlights amitriptyline’s superior functionality improvement.

Contrarily, Gammoh et al. (2021) report pregabalin as more effective than GBP, Romanò et al. (2009) see added benefits with pregabalin combined with celecoxib but only in cases where both nociceptive and neuropathic elements were present, and Saldaña et al. (2010a) note its cost-effectiveness in clinical practice.

Some previous meta-analyses, as noted by Wewege et al. (2023), did not specifically focus on pregabalin but considered a wider array of medications, complicating the isolation of variables that could influence outcomes. Another meta-analysis by Giménez-Campos et al. (2022) included only three randomised controlled trials (RCTs) for pregabalin, and they did not account for dosage, controls, and other confounding variables. Baron et al. (2010) concluded that “further work” was required to understand the potential of pregabalin in treating LBP. These insights underscore the necessity for a new meta-analysis aimed at objectively assessing the therapeutic value of pregabalin, addressing the gaps identified in earlier studies.

This meta-analysis addresses gaps by providing an up-to-date, focused synthesis of studies that evaluate both the efficacy and safety of pregabalin specifically in non-surgical LBP. Through rigorous inclusion criteria and the integration of recent data, this work aims to comprehensively assess the efficacy and safety of pregabalin in the management of LBP, addressing gaps in current research and providing clear, evidence-based insights to guide clinical practice.

## 2 Materials and methods

### 2.1 Eligibility criteria

The study protocol has been registered with PROSPERO (CRD42025642429) and follows the Preferred Reporting Items for Systematic Reviews and Meta-Analyses (PRISMA) guidelines (Page et al., 2021). PICOS strategy was used as follows: Patients (P): adult patients diagnosed with low back pain, radiculopathy, or spinal stenosis, the Intervention (I) under scrutiny was the administration of pregabalin, which was Compared (C) against placebos or alternative pain medications; the primary Outcomes (O) assessed were the efficacy and safety of the treatments and the analysis was strictly confined to data extracted from Studies (S) that were comparative studies.

The exclusion criteria were rigorously applied as follows: duplicates were excluded to prevent data redundancy and potential analytical bias. The focus on adult populations led to the exclusion of non-adult studies, ensuring consistency in pharmacological responses and treatment effects. Studies involving patients who received surgery were also excluded. Additionally, studies with incomplete or missing data, or those that failed to report on all relevant variables, were excluded to enhance the robustness and accuracy of the statistical analysis.

### 2.2 Information sources and search methods for identification of studies

The literature search was conducted on December 2024 across PubMed, Scopus, and the Cochrane Library without restrictions on publication date or language. The search strategy employed keywords such as “Pregabalin,” “Lyrica,” “low backache,” “radiculopathy,” “spinal stenosis,” “neurogenic claudication,” “sciatica,” “low back pain,” “lumbalgia,” “lumbar,” and “spine,” detailed further in Supplementary File S1. Additionally, a manual search of references was performed to ensure comprehensive coverage. Two reviewers independently screened the studies, and any discrepancies or disagreements were resolved by involving a third reviewer.

### 2.3 Data extraction and data items

Data extraction was carried out by two reviewers, with any disagreements resolved by a third reviewer. They meticulously gathered baseline characteristics, including study details, region, period, type of study, follow-up duration, patient count, age, number of females, patient demographics, and detailed intervention information. Treatment regimens and dosages were also extensively documented. The primary outcomes measured included pain, assessed using the Visual Analogue Scale (VAS) for back and leg pain, Brief Pain Inventory and Pain Rating Index; anxiety and depression, evaluated with the Hospital Anxiety and Depression Scale (HADS); disability, assessed by the Oswestry Disability Index (ODI), Roland-Morris Disability Questionnaire, and Sheehan Disability Inventory; sleep disturbances, measured using the Daily Sleep Interference Scale, Insomnia Severity Index (ISI), Medical Outcomes Study Sleep Scale (MOS), and Pain-Related Sleep Interference Scale (PRSIS); and quality of life, evaluated using the EuroQol-5 Dimension (EQ-5D) health status index and the Short Form-12 (SF-12). Adverse events and costs were also systematically recorded.

### 2.4 Assessment of risk of bias in included studies

The assessment of the risk of bias was conducted using the Cochrane Collaboration’s tool (RoB 2) and implemented through Review Manager software (RevMan, version 5.4.1, The Cochrane Collaboration, London, United Kingdom) for studies that were RCTs. This analysis encompassed six domains: random sequence generation, allocation concealment, blinding of participants and personnel, blinding of outcome assessment, incomplete outcome data, and selective reporting. Each study was evaluated for these criteria and categorized as having a low, high, or unclear risk of bias based on how well they addressed these key aspects of trial integrity.

The methodological quality of the comparative studies that were non-RCTs were independently evaluated by two reviewers using the Methodological Index for Non-Randomized Studies (MINORS) criteria (Slim et al., 2003). This instrument contains items to evaluate key methodological aspects such as study design, patient selection, outcome measures, and follow-up. Scoring ranges from 0 to 24 for comparative designs. Comparative studies scoring 0–6 will be deemed very low-quality, 7–10 as low quality, 11–15 as fair quality, and 16–24 as high quality. Any discrepancies in the quality assessment scoring between the two reviewers were discussed to reach a consensus.

### 2.5 Assessment of results

The statistical analyzes were conducted using RevMan version 5.4.1 Dichotomous variables were analyzed using odds ratios (ORs) with 95% confidence intervals (CIs), while continuous variables were assessed through mean differences (MDs) and 95% CIs. For continuous variables with non-compatible units or scales, standardized mean differences (SMDs) and 95% CIs were calculated. In this meta-analysis, positive or negative values of MD and SMD were used to indicate the direction of effect, depending on whether the outcome favored the intervention (pregabalin) or the control. A negative value indicates an effect in favor of pregabalin when lower scores are associated with better outcomes, while positive values reflect outcomes favoring the control or indicating increased values in the pregabalin group. Heterogeneity among study results was evaluated using the chi-square statistic and the I^2^ test, with I^2^ values ranging from 0% to 100% indicating low, moderate, and high heterogeneity at values of 25%, 50%, and 75%, respectively (Lorente et al., 2024). A fixed-effects model was applied in cases of no significant heterogeneity, and a random-effects model was used when heterogeneity was substantial (I^2^ ≥ 50%). For extracting data from figures, WebPlotDigitizer version 4.5 (Automeris, Pacifica, California, United States) was employed. Any missing data were addressed following guidelines from the Cochrane Handbook, ensuring a rigorous and methodical approach to data synthesis and interpretation (Higgins et al., 2019).

### 2.6 Publication bias

The assessment of publication bias in the analysis was conducted using Review Manager version 5.4.1 software. To detect any asymmetry indicative of bias, funnel plots were generated and subjected to careful visual inspection. This method involves plotting the treatment effects from individual studies against a measure of study size or precision, allowing for the identification of any systematic deviations from the expected distribution.

### 2.7 Additional analyses

Subgroup analyzes were conducted when the dataset included a sufficient and statistically robust number of studies, ensuring the reliability of the findings. These analyzes included assessments based on different follow-up periods: 2, 4, 6, 8, and more than 8 weeks. Additionally, a subgroup of patients treated with pregabalin plus another intervention was examined to assess the combined effect of pregabalin separately.

Sensitivity analyzes were conducted by excluding the study with the greatest weight to test the robustness of the results.

The certainty of the results was evaluated using the Grading of Recommendations Assessment, Development, and Evaluation (GRADE) framework in GRADEpro, which considers study design, risk of bias, inconsistency, indirectness, imprecision, and publication bias (Guyatt et al., 2013).

## 3 Results

### 3.1 Study selection

**FIGURE 1:**
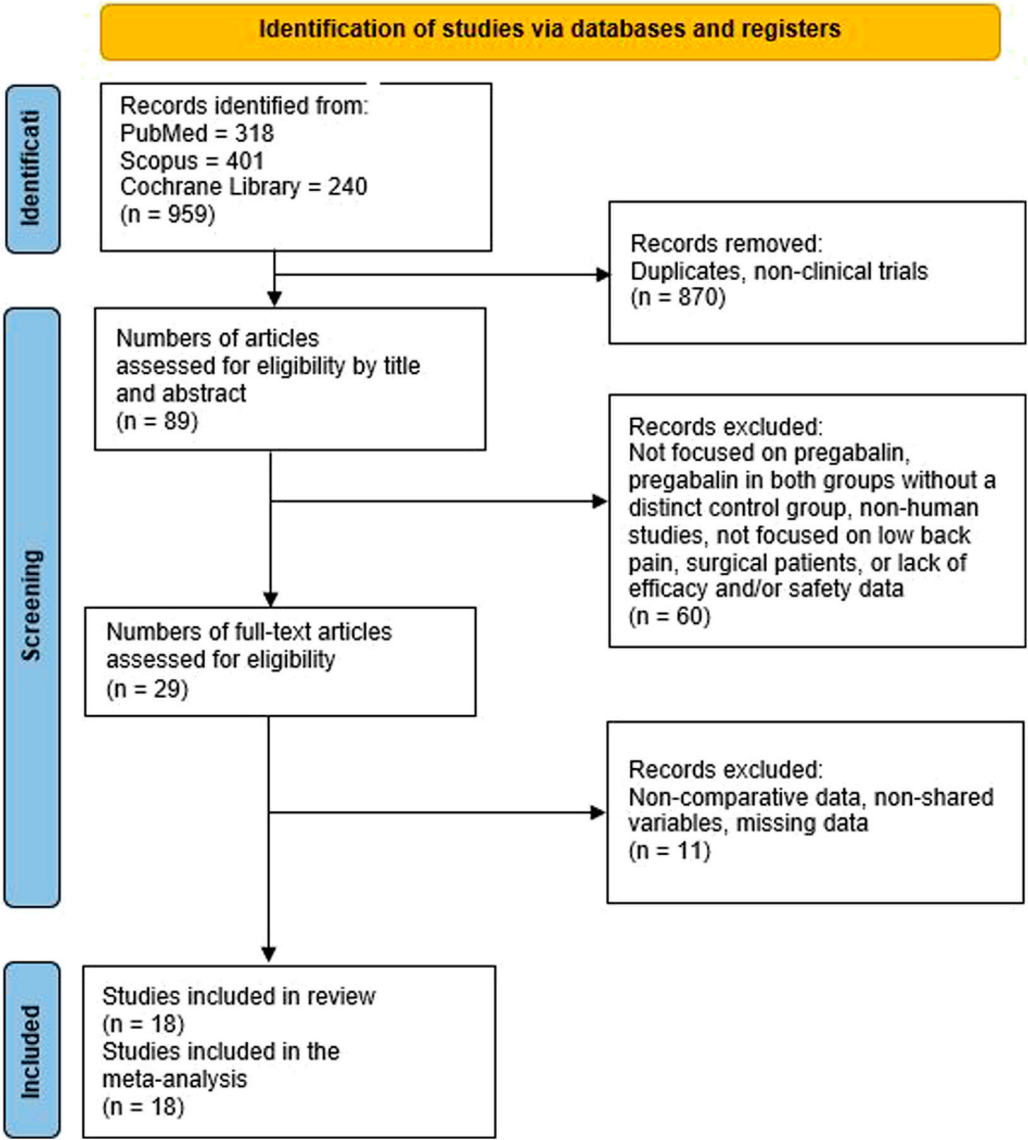
PRISMA flowchart illustrating the study selection process for the systematic review and meta-analysis.

The initial search in the databases yielded 959 studies. After adjusting for duplicates and clinical trials, 870 studies were excluded, leaving 89 studies. Upon reviewing the titles and abstracts, 60 studies were excluded for the following reasons: they did not include pregabalin, they involved pregabalin in both groups without a distinct control group, they were not studies on humans, they did not focus on low back pain, they included surgical patients, or they lacked efficacy and/or safety data. After a full-text review of the remaining studies, 11 were further excluded due to missing data, non-comparable data, or inconsistent variables, resulting in a total of 18 studies eligible for inclusion. A manual review of the references from these studies did not yield any additional studies to include. Ultimately, 18 studies were included in the meta-analysis (Figure 1; Table 1) (Morera-Domínguez et al., 2010; Robertson et al., 2019; Sakai et al., 2015; Mathieson et al., 2017; Park et al., 2024; Kalita et al., 2014; Gammoh et al., 2021; Romanò et al., 2009; Saldaña et al., 2010a; Baron et al., 2010; Baron et al., 2014; Chye et al., 2021; Kim et al., 2016; Markman et al., 2015; Pota et al., 2012; Saldaña et al., 2010b; Sicras-Mainar et al., 2013; Taguchi et al., 2015; Yeole et al., 2022).

**TABLE 1:** Baseline characteristics of the included studies.

Study	Region	Period	Follow-up	n PGB/active control or placebo/combination	Age PGB/active control or placebo/combination	Female PGB/active control or placebo/combination	Type of patients	Intervention
Baron et al. (2010)	Belgium, Canada, Germany, Italy, Spain, Sweden, Turkey, and the United States	2005–2007	70 days	110/107	52.5/52.6	54/59	Chronic lumbosacral radiculopathy due to spinal stenosis or disk herniation	Pregabalin 150–600 mg/day during 4 weeks versus placebo
Baron et al. (2014)	Germany	NR	8 weeks	159/154	56.3/58.5	86/95	Chronic low back pain lasting ≥3 months prior to enrollment	Pregabalin from 150 mg/day to 300 mg/day + tapentadol PR 300 mg/day versus tapentadol PR 400 mg/day to 500 mg/day
Chye et al. (2021)	Australia	NR	52 weeks	94/91	53.3/55	60/45	Moderate to severe sciatica present for at least 1 week and at most 1 year	Pregabalin with a starting dose of 150 mg per day, adjusted to a maximum dose of 600 mg versus placebo
Gammoh et al. (2021)	Jordan	NR	6 weeks	40/44	NR	18/20	Neuropathic low back pain	Pregabalin from 75 mg/day to 300 mg/day versus gabapentin from 400 mg/day to 800 mg/day
Kalita et al. (2014)	India	NR	14 weeks	97/103	42.0/41.6	NR	Chronic low backache: localized, with radiculopathy or with lumbar canal stenosis. Due ot nerve root compression; disc protrusion and lumbar canal stenosis	Pregabalin 75 mg twice daily for 2 weeks, followed by 150 mg twice daily for 4 weeks and then 300 mg twice daily. Amitriptyline 12.5 mg for 2 weeks followed by 25 mg for 4 weeks and then increased to 50 mg
Kim et al. (2016)	Korea	NR	8 weeks	60/61/61	61.4/62.9/62.0	42/42/38	Lumbar spinal stenosis (Mean of symptom duration: more than 5 months)	Pregabalin, 75 mg 3 times per day; limaprost, 5 μg 3 times per day; combination of limaprost and pregabalin, 3 times per day
Markman et al. (2015)	USA	2008–2010	14 days	14/15	71.1/69	4/5	Lumbar spinal stenosis and symptoms of neurogenic claudication for 3 months	Pregabalin from 150 mg/day to 300 mg/day versus placebo
Mathieson et al. (2017)	Australia	NR	8 weeks	106/101	52.4/55.2	66/49	Moderate to severe sciatica (mean of leg pain: more than 2 months)	Pregabalin from 150 mg/day to 600 mg/day versus placebo
Morera-Domínguez et al. (2010)	Spain	2006–2007	12	564/119	56.3/54.7	256/53	Low back pain: had pain secondary to lumbosacral radiculopathy, lumbar or sacral pain, irradiating to the calves or feet, and exhibiting a distribution consistent with involvement of nerve root L5 or S1	Pregabalin (mean dose 189 ± 141.7 mg/day), versus those who modified or added to their previous treatment an analgesic other than pregabalin
Park et al. (2024)	Korea	2020–2023	6 weeks	109/108	66.3/65.1	61/60	Lumbar spinal stenosis	Pregabalin 150 mg/day, versus limaprost 15 mg/day
Pota et al. (2012)	Italy	NR	6 weeks	22/22	NR	NR	Chronic mechanical degenerative low back pain	Buprenorphine 35 μg/h plus pregabalin 300 mg/day (two 150 mg tablets) versus buprenorphine 35 μg/h plus placebo
Robertson et al. (2019)	USA	2016–2018	8 weeks	10/8	57/57	0/0	Unilateral chronic sciatica, imaging corroborating a root level lesion concordant	First PGB 150 mg–300 mg and GBP (400–800 mg) for 8 weeks, after it is changed
Romanò et al. (2009)	Italy	2006–2008	4 weeks	12/12/12	53	20	Chronic low-back pain (symptoms duration: [6 months, mean: 13 ± 6 months) due to disc prolapse, lumbar spondylosis, and/or spinal stenosis	Pregabalin (approximately 1 mg/kg/day the first week and then 2–4 mg/kg/day); Celecoxib (approximately 3–6 mg/kg/day); Celecoxib (approximately 3–6 mg/kg/day) plus pregabalin (approximately 1 mg/kg/day the first week and then 2–4 mg/kg/day)
Sakai et al. (2015)	Japan	2011–2012	4 weeks	30/30	72.03/72.60	9/11	Chronic low back pain	75 mg pregabalin or tramadol/acetaminophen combination tablets (TRAM/APAP) twice daily doses
Saldaña et al. (2010b)	Spain	2005–2006	12 weeks	473/155/676	56.0/56.3/57.2	262/90/377	Refractory painful radiculopathy of cervical or lumbosacral origin	PGB Monotherapy (mean dose was 187 ± 106 mg/day) and combination of other drugs and PGB treatment, add-on (mean dose of PGB 191 ± 107 mg/day)
Sicras-Mainar et al. (2013)	Spain	2006/2008	12 months	378/193	60.1/58.6	NR	Painful axial radiculopathy: syndromes related to the compression of peripheral nerves or roots (lumbar, thoracic or cervical radiculopathy	Treatment during more than 5 months of pregabalin (150–600 mg/day) or gabapentin (dose from 900 to 3,600 mg/day)
Taguchi et al. (2015)	Japan	2014	8 weeks	157/174	69.1/70.7	92/106	Chronic low back pain with accompanying neuropathic pain	Pregabalin dosing was flexible, and the range of doses among these patients was 25 mg/day to 300 mg/day versus usual care
Yeole et al. (2022)	India	NR	8 weeks	160/159	43.13/45.26	98/94	Chronic lower back pain having at least one of the following five features on the side corresponding to leg pain: (a) sharp and shooting pain below the knee; (b) pain evoked by straight leg raising to 60_ or less; (c) decreased or absent ankle reflex; (d) weakness of muscles below the knee; (e) sensory loss in L5/S1 distribution, with a pain score of at least 4 on the numeric rating scale (NRS), without any critical illness or medical conditions	Pregabalin prolonged release (75 mg) and etoricoxib (60 mg) in comparison to etoricoxib (60 mg) alone during 8 weeks

NR, not reported.

### 3.2 Risk of bias

**FIGURE 2:**
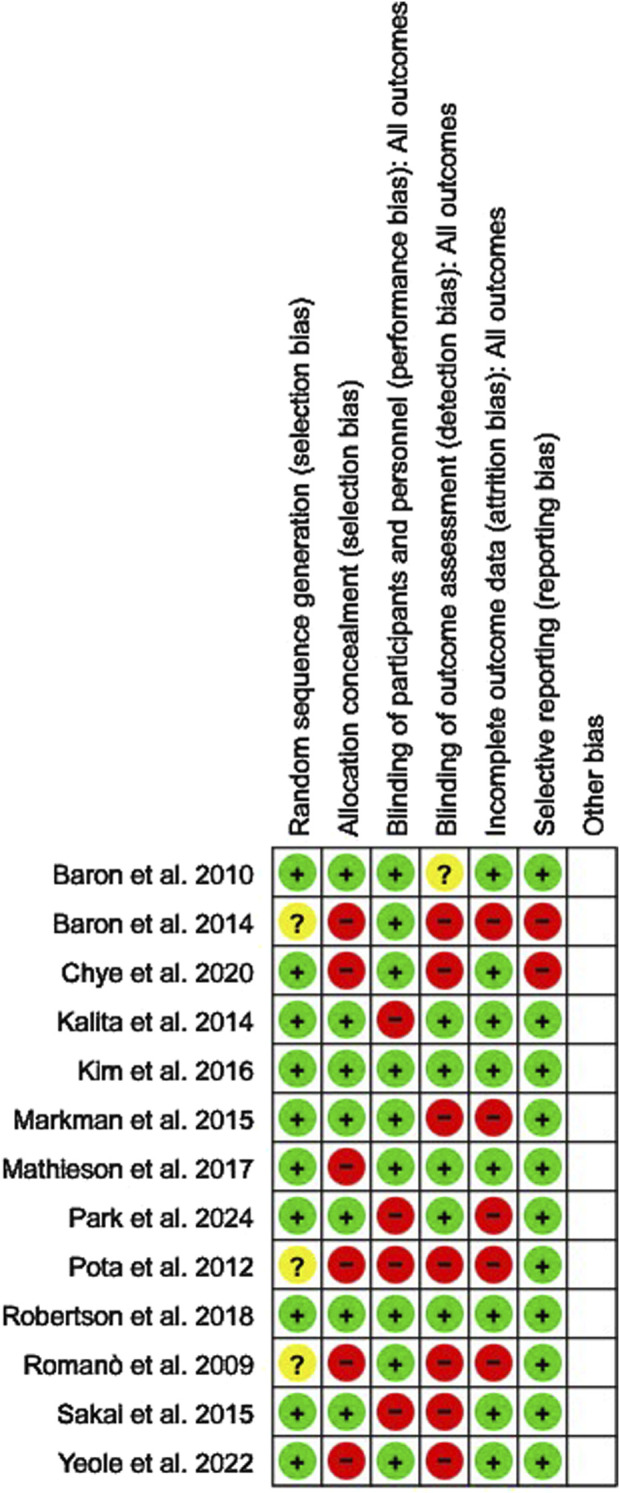
Risk of bias assessment of randomized controlled trials (RCTs) included in the meta-analysis, conducted using the Cochrane Collaboration’s RoB 2 tool.

The overall and individualized risks of bias for the included RCTs studies are shown in Figure 2. These studies demonstrated a low risk of bias regarding reporting bias and random sequence generation. There was a moderate risk of bias for incomplete outcome data, allocation concealment and patient and personnel blinding and high risk of bias of blinding of outcome assessment.

The quality of the included non-RCT studies, as assessed by the MINORS tool and deemed to have high quality, is shown in Supplementary Table S1.

### 3.3 Study characteristics

Table 1 presents the basic characteristics of the studies included. Eighteen studies and 5,000 patients were included. Most of the studies were from Europe and Asia (seven studies each). The mean age ranged from 42.0 to 72.6 years in the pregabalin group. The number of female participants, the type of patients and interventions are presented in Table 1. The therapeutic regimens are shown in Supplementary Table S2.

### 3.4 Outcomes

#### 3.4.1 Pain assessment

**FIGURE 3:**
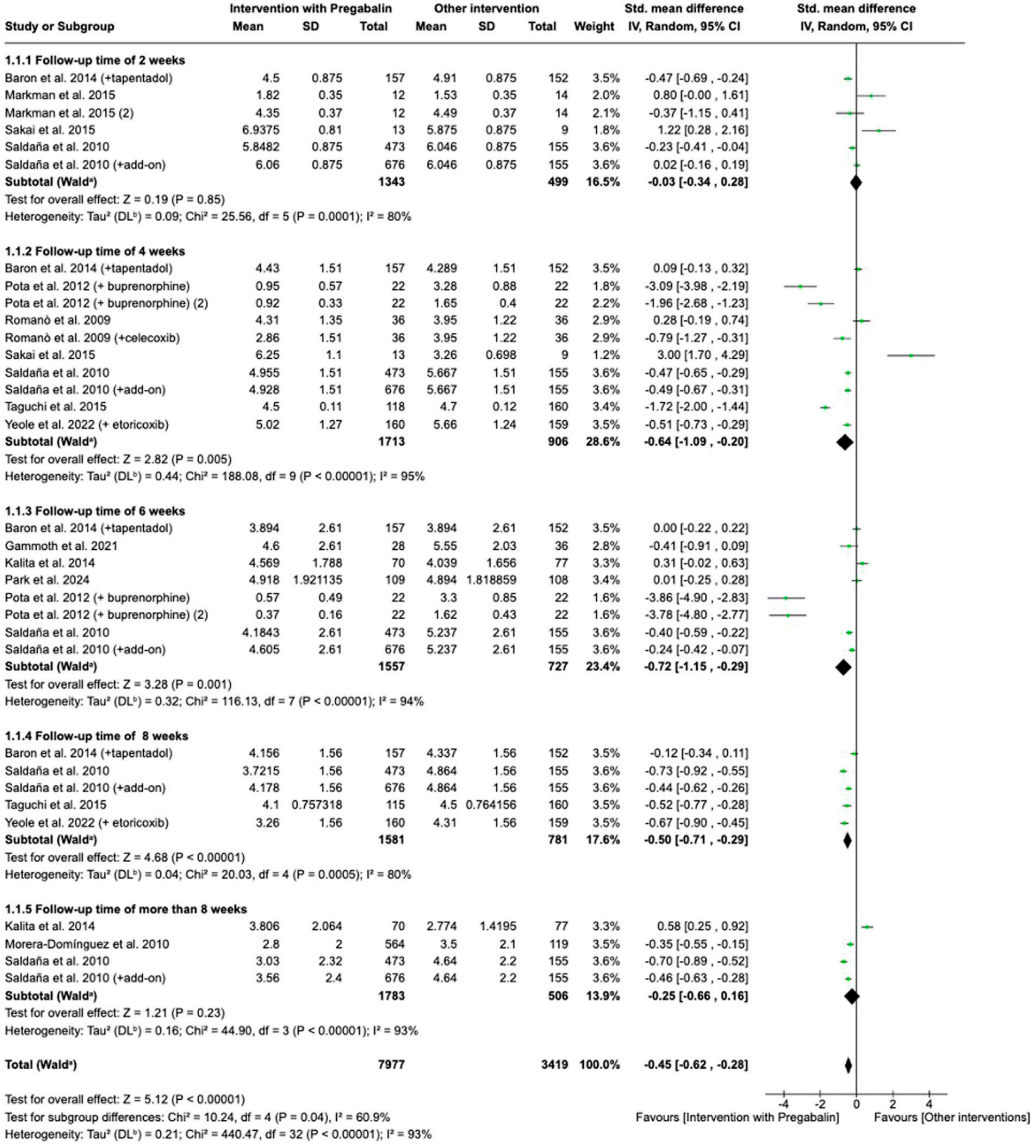
Forest plot of standardized mean differences (SMDs) for back pain intensity (Visual Analogue Scale) comparing pregabalin versus control interventions across follow-up periods: 2 weeks; 4 weeks; 6 weeks; 8 weeks; and >8 weeks. Squares represent study-specific effect sizes (SMD), with horizontal lines indicating 95% confidence intervals. Study of Saldaña et al. (2010a) refers to Saldaña et al. (2010b).

LBP assessed and VAS scale was studied according to different follow-up times (Figure 3). Attending the shortest follow-up time, at 2 weeks, pregabalin or combination with pregabalin (tapentadol or other combination) did not show better results in reducing pain intensity when it is compared to active control group (diphenhydramine, tapentadol, tramadol/acetaminophen or other drug combination) (SMD = −0.03, 95% CI = −0.34 to 0.28, participants = 1,842, studies = 4, I^2^ = 80%; p = 0.85). However, pregabalin or combination with pregabalin (combination with tapentadol or buprenorphine, etoricoxib, celecoxib, among others) significantly improved pain level at 4 weeks of follow-up when it is compared to active controls (tapentadol, tramadol/acetaminophen, buprenorphine, etoricoxib, celecoxib, other drug combination or usual care treatment) (SMD = −0.64, 95% CI = −1.09 to −0.20, participants = 2,619, studies = 7, I^2^ = 95%, p = 0.005). Similar results were obtained when pregabalin or combination with pregabalin (combination with tapentadol or buprenorphine, mainly) were analyzed versus other interventions at 6 weeks (SMD = −0.72, 95% CI = −1.15 to −0.29, participants = 2,284, studies = 6, I^2^ = 94%, p = 0.001) and 8 weeks (SMD = −0.50, 95% CI = −0.71 to −0.29, participants = 2,284, studies = 6, I^2^ = 80%, p = 0.0005) Three studies analyzed pain level at follow-up times of more than 8 weeks. Morera-Domínguez et al. (2010) and Saldaña et al. (2010b) performed the analysis at 12 weeks of follow-up and Kalita et al. (2014) at 14 weeks from the start of intervention. In this case, there were no significant differences from active controls (SMD = −0.25, 95% CI = −0.66 to 0.16, participants = 2,289, studies = 3, I^2^ = 93%, p = 0.23).

Four studies analyzed effects of pregabalin on leg pain through VAS scale (Robertson et al., 2019; Mathieson et al., 2017; Park et al., 2024; Chye et al., 2021). When effect of pregabalin or combination with pregabalin was compared to alternative on leg pain, there were no significant differences (MD = 0.08, 95% CI = −0.17 to 0.33, participants = 624, studies = 4, I^2^ = 53%, p = 0.54).

#### 3.4.2 Anxiety and depression

**FIGURE 4:**
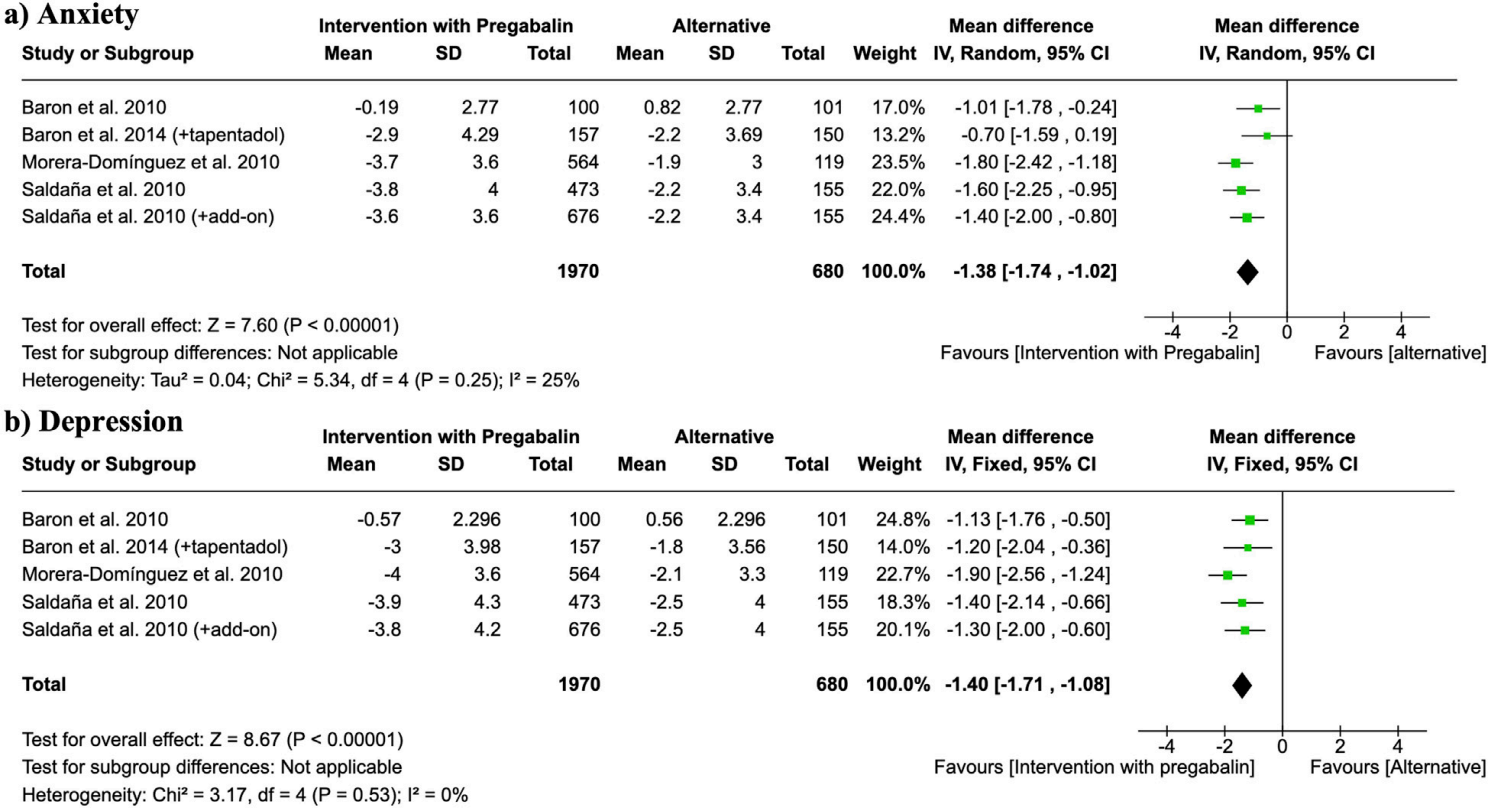
Forest plots of mean differences (MDs) for **(a)** anxiety and **(b)** depression scores (measured by Hospital Anxiety and Depression Scale, HADS) comparing pregabalin interventions versus control groups. Study of Saldaña et al. (2010a) refers to Saldaña et al. (2010b).

Hospital Anxiety and Depression Scale (HADS) were used to analyze anxiety and depression by the authors. An intervention with pregabalin showed an improvement of anxiety levels at different endpoints of follow-up compared to active control group (placebo, other analgesic than pregabalin, tapentadol) (MD = −1.38, 95% CI = −1.74 to −1.02, participants = 2,650, studies = 3, I^2^ = 25%, p < 0.00001) (Figure 4A). The same studies analyzed depression levels through HADS test. Pregabalin presented significantly less depressive symptoms (MD = −1.40, 95% CI = −1.71 to −1.08, participants = 2,650, studies = 3, I^2^ = 0%, p < 0.00001) (Figure 4B).

#### 3.4.3 Quality of life, disability

**FIGURE 5:**
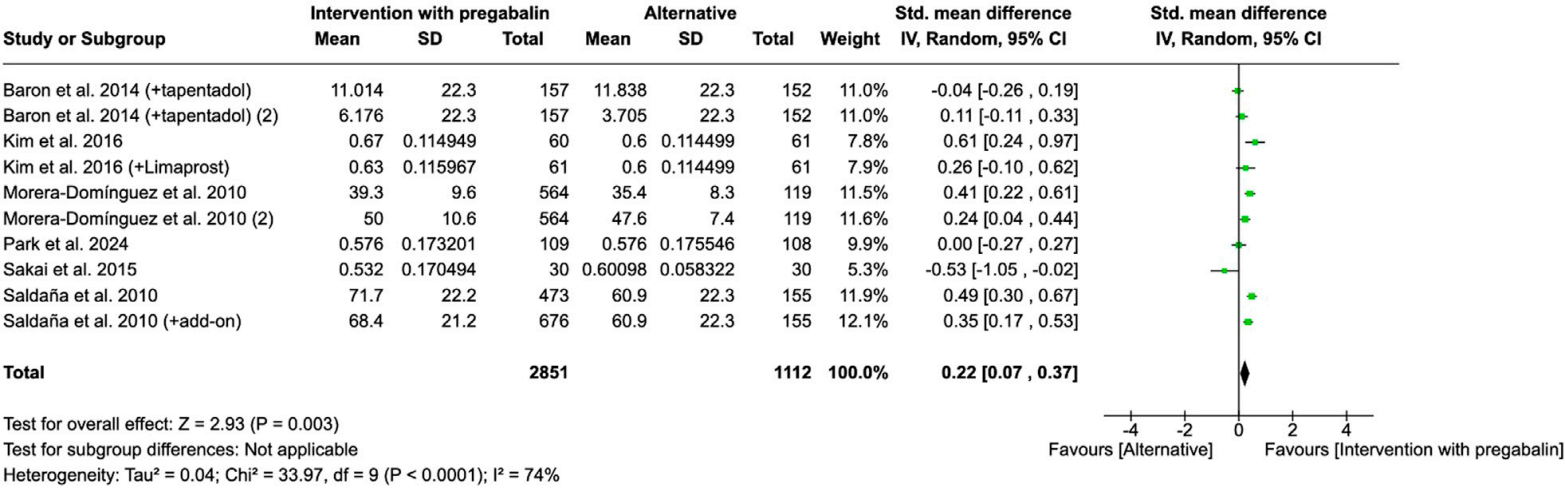
Forest plot of standardized mean differences (SMDs) for quality of life outcomes (measured by EQ-5D or SF-12) comparing pregabalin interventions versus control groups. Study of Saldaña et al. (2010a) refers to Saldaña et al. (2010b).

Quality of life was studied by six studies through EQ-5D and SF-12 tests. Forest plot showed an improvement of quality of life when patients were treated with pregabalin compared to control group (tapentadol, limaprost, other analgesic than pregabalin, tramadol/acetaminophen combination, or placebo) (SMD = 0.22, 95% CI = 0.07 to 0.37, participants = 3,963, studies = 6, I^2^ = 74%, p = 0.003) (Figure 5).

**FIGURE 6:**
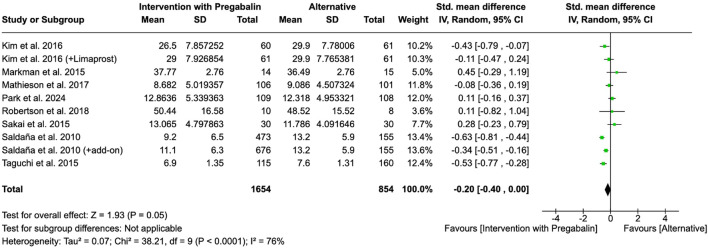
Forest plot of standardized mean differences (SMDs) for disability outcomes comparing pregabalin interventions versus control groups. Study of Saldaña et al. (2010a) refers to Saldaña et al. (2010b).

The effect of pregabalin on the improvement of disability was studied by eight of the included studies. Authors used different scales like ODI, Roland-Morris Disability Questionnaire or Sheehan Disability Inventory. An intervention with pregabalin did not significantly improve disability levels (SMD = −0.20, 95% CI = −0.40 to 0.00, participants = 2,508, studies = 8, I^2^ = 76%, p = 0.05) (Figure 6).

#### 3.4.4 Sleep disturbance

**FIGURE 7:**
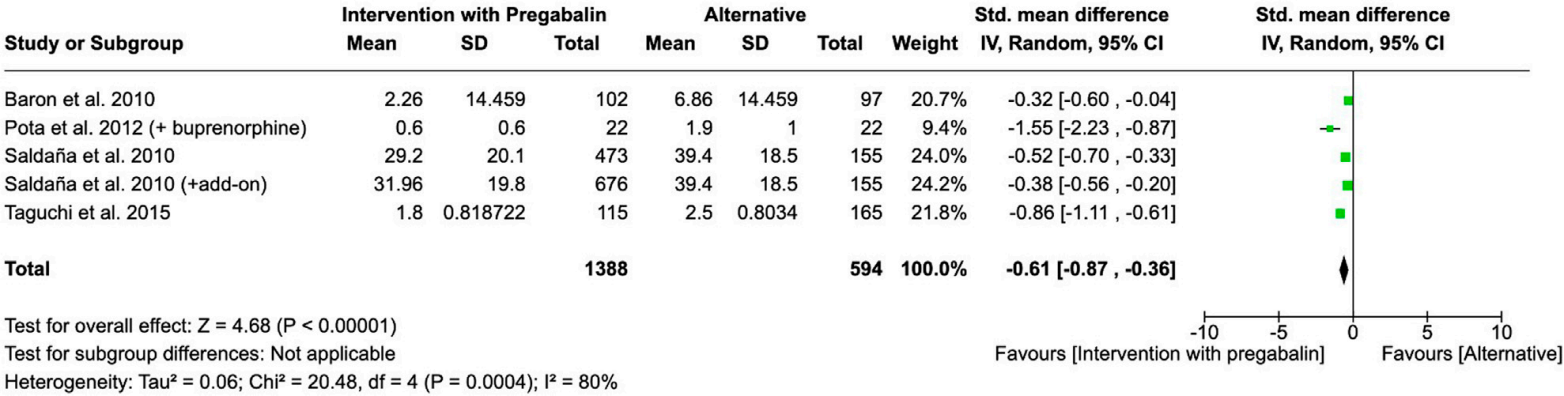
Forest plot of standardized mean differences (SMDs) for sleep disturbance outcomes comparing pregabalin interventions versus control groups. Study of Saldaña et al. (2010a) refers to Saldaña et al. (2010b).

Four studies analyzed the effect of an intervention with pregabalin versus buprenorphine, other analgesic, or placebo on sleep disturbance. An intervention with ‘pregabalin (buprenorphine and pregabalin, other analgesics and pregabalin, or pregabalin alone) showed a reduction on sleep disturbance when it was compared to alternative treatments or placebo (SMD = −0.61, 95% CI = −0.87 to −0.36, participants = 1,982, studies = 4, I^2^ = 80%, p < 0.00001) (Figure 7).

#### 3.4.5 Adverse events

There were no significant differences with respect to the adverse events assessed by five of the included studies: dizziness, nausea, drowsiness, bowel disturbance, and edema (Table 2), in the case where the treatment involved a combination of other medications with pregabalin (buprenorphine, limaprost, or GBP) and whether the intervention was pregabalin alone versus an active control.

**TABLE 2:** Adverse effect assessment.

Effect size	n participants	Random/fixed effect model (OR 95% CI)	I^2^ (%)	*P*-value
Dizziness				
PGB or Combination with PGB vs. alternative	688	OR = 1.72, 95% CI = 0.66–4.51	53	0.27
PGB vs. alternative	583	OR = 2.23, 95% CI = 0.79–6.29	52	0.13
Combination with PGB vs. alternative	166	OR = 0.67, 95% CI = 0.04–10.51	62	0.77
Nausea				
PGB/Combination with PGB vs. alternative	264	OR = 1.69, 95% CI = 0.51–5.61	0	0.39
PGB vs. alternative	159	OR = 2.71, 95% CI = 0.44–16.50	0	0.28
Combination with PGB vs. alternative	166	OR = 1.00, 95% CI = 0.18–5.60	N/A	1.00
Drowsy				
PGB/Combination with PGB vs. alternative	481	OR = 0.82, 95% CI = 0.33–2.05	22	0.67
PGB vs. alternative	332	OR = 0.80, 95% CI = 0.08–7.87	53	0.85
Combination with PGB vs. alternative	166	OR = 1.00, 95% CI = 0.27–3.65	0	1.00
Bowel disturbance				
PGB/Combination with PGB vs. alternative	264	OR = 2.27, 95% CI = 0.64–8.09	0	0.20
PGB vs. alternative	159	OR = 3.11, 95% CI = 0.34–28.22	0	0.31
Combination with PGB vs. alternative	166	OR = 1.86, 95% CI = 0.39–8.99	N/A	0.44
Edema				
PGB/Combination with PGB vs. alternative	399	OR = 1.34, 95% CI = 0.30–6.04	0	0.70
PGB vs. alternative	338	OR = 2.37, 95% CI = 0.34–16.24	0	0.38
Combination with PGB vs. alternative	122	OR = 0.33, 95% CI = 0.01–8.21	N/A	0.50

CI, confidence interval; N/A, not applicable; OR, odds ratio; PGB, pregabalin.

#### 3.4.6 Total cost

Total cost of a treatment with pregabalin versus placebo or other treatments was analyzed by four studies. This analysis showed that a treatment with pregabalin resulted in lower total costs per patient at the end of the various follow-up periods (from 12 to 52 weeks) of each study (SMD = −0.16, 95% CI = −0.25 to −0.07, participants = 2,898, studies = 4, I^2^ = 9%, p = 0.0003).

#### 3.4.7 Sensitivity analyses

Sensitivity analyzes were conducted by excluding the study with the greatest weight to test the robustness of the results obtained on pain, anxiety and depression, quality of life, sleep, disability and total cost outcomes. Results did not change significantly, except in the case of follow-up time of 4 weeks when back pain was assessed where, upon removing the study with the greatest weight, the significance was lost and no beneficial effects were observed at this period of treatment with pregabalin versus an active control (SMD = −0.66, 95% CI = −1.36 to 0.04, participants = 1,080, studies = 6, I^2^ = 96%, p = 0.07).

### 3.5 Publication bias

Upon visual inspection of the funnel plots, asymmetry was observed indicating publication bias in most of the variables. Symmetry was observed in case of leg pain, depression assessment, total cost, and for some of the adverse events (nausea, drowsiness, bowel disturbance, and edema).

### 3.6 GRADE

The GRADE assessment of the main outcomes (back pain, anxiety, depression, disability, quality of life and sleep) is presented in Table 3. The certainty of anxiety and the back pain outcomes was low, whereas depression, quality of life, sleep, and disability outcomes were moderate. The studies mainly presented a high risk of publication bias, differences in inclusion criteria and four of the included studies had moderate or high risk of bias.

**TABLE 3:** GRADE assessment.

Certainty assessment							№ of patients		Effect		Certainty	Importance
№ of studies	Study design	Risk of bias	Inconsistency	Indirectness	Imprecision	Other considerations	[Intervention with pregabalin]	[Placebo or other interventions]	Relative (95% CI)	Absolute (95% CI)		
Back pain assessment												
12	Randomised trials	Serious^a^	Not serious	Serious^b^	Not serious	Publication bias strongly suspected strong association^c^	7,921	2,564	-	SMD 0.45 SD lower (0.62 lower to 0.28 lower)	⊕⊕○○ Low^a^ ^,b,c^	Critical
Anxiety												
4	Randomised trials	Not serious	Not serious	Serious^b^	Not serious	Publication bias strongly suspected^c^	1,970	525	-	MD 1.38 lower (1.74 lower to 1.02 lower)	⊕⊕○○ Low^b,c^	Critical
Depression												
4	Randomised trials	Not serious	Not serious	Serious^b^	Not serious	None	1,970	525	-	MD 1.4 lower (1.71 lower to 1.08 lower)	⊕⊕⊕○ Moderate^b^	Critical
Disability												
8	Randomised trials	Not serious	Not serious	Serious^b^	Not serious	Publication bias strongly suspected strong association^c^	1,654	699	-	SMD 0.2 SD lower (0.4 lower to 0)	⊕⊕⊕○ Moderate^b^ ^,^ ^c^	Critical
Quality of life												
6	Randomised trials	Not serious	Not serious	Serious^b^	Not serious	Publication bias strongly suspected strong association^c^	2,130	686	-	SMD 0.22 SD higher (0.07 higher to 0.37 higher)	⊕⊕⊕○ Moderate^b^ ^,^ ^c^	Important
Sleep												
4	Randomised trials	Not serious	Not serious	Serious^b^	Not serious	Publication bias strongly suspected strong association^c^	1,388	439	-	SMD 0.61 SD lower (0.87 lower to 0.36 lower)	⊕⊕⊕○ Moderate^b^ ^,^ ^c^	Important

^a^ High proportion of included studies with moderate or high risk of bias.

^b^ Differences in inclusion criteria.

^c^ Publication bias detected through the Funnel Plots.

CI, confidence interval; MD, mean difference; SMD, standardised mean difference.

## 4 Discussion

Despite the development of multiple drugs targeting diverse mechanisms of action for predominantly nonspecific axial LBP syndromes, no therapy has been approved for neuropathic LBP conditions such as sciatica (Markman et al., 2018). Pregabalin has shown efficacy in several neuropathic and nociplastic pain conditions, including diabetic peripheral neuropathy (Zhang et al., 2015; Parsons et al., 2018) and fibromyalgia (Zareba, 2008; Migliorini et al., 2022). However, evidence remains inconsistent for other conditions, such as LBP, particularly in neuropathic subtypes like sciatica.

Pregabalin has demonstrated improvements in LBP at 4, 6, and 8 weeks, underscoring the importance of addressing the multidimensional nature of this condition, which can involve both neuropathic and nociceptive pain components. Notably, our meta-analysis indicates that the most pronounced effectiveness of pregabalin emerges from 4 weeks onward, which contrasts with findings for other compounds such as buprenorphine (Pota et al., 2012) or conventional care (Taguchi et al., 2015), where the onset of pain relief may differ. In addition to its analgesic effects, pregabalin has shown efficacy in improving reduction in sleep disturbance and anxiety symptoms, which are frequently associated with chronic LBP (Montaño et al., 2025). This psychiatric benefit may help explain the significant improvements in quality of life observed, even in the absence of marked changes in functional or disability scores. It is important to note that many of the included studies had relatively short follow-up durations (≤12 weeks), which may have limited the detection of changes in disability outcomes. Improvements in sleep quality associated with pregabalin may contribute to overall symptom relief, as a strong relationship between sleep quality and quality of life has been documented (Perotta et al., 2021).

In conditions such as sciatica and radiculopathy, pulsed radiofrequency has demonstrated efficacy in pain relief (Chao et al., 2008) both as a standalone treatment and when combined with steroid injection (Napoli et al., 2023). However, these approaches have not yet been specifically evaluated in combination with pregabalin for these indications. Interestingly, the combination of pregabalin with radiofrequency has been shown to improve sleep quality in patients with herpetic neuralgia, further supporting its beneficial effects on overall patient wellbeing (Chen et al., 2023). These findings suggest that exploring combined treatment approaches could be valuable for enhancing outcomes in patients with neuropathic pain conditions.

The heterogeneity of comparators in existing studies—including gabapentin (GBP), amitriptyline, limaprost, diphenhydramine, NSAIDs, and opioids—has limited the number of trials directly comparing pregabalin to individual agents, constraining robust conclusions. However, the literature indicates that pregabalin showed superiority over gabapentin in cancer-related neuropathic pain (Mishra et al., 2012) and over opioids for lower-limb symptoms in elderly patients with chronic LBP (Sakai et al., 2015).

In this meta-analysis, the combination of pregabalin with other pharmacological agents demonstrated a significant improvement in pain outcomes compared to active control or placebo. These findings suggest that adjunctive pregabalin may represent an optimal strategy for enhancing pain management in patients with lower back pain, particularly when a neuropathic component is present (Romanò et al., 2009). It is important to note that certain combinations, such as pregabalin with opioids, have been associated with increased risks of sedation and falls (Virnes et al., 2022) as well as dizziness, cognitive dysfunction, and respiratory depression (Hahn et al., 2022).

Another important question to address is regarding the comparative effectiveness and safety of higher doses of pregabalin versus alternative regimens. Future research should categorize pregabalin dosing (e.g., ≤150 mg, 150–300 mg, >300 mg) to better elucidate potential dose–response relationships. In our meta-analysis, only three studies explicitly reported mean doses above 300 mg; however, this does not necessarily indicate that all patients received doses exceeding 300 mg, but rather that the average dose within those studies was higher. Similarly, the initial starting dose did not appear to influence outcomes, as studies commencing pregabalin at 75 mg and those starting between 150 and 300 mg achieved comparable results, provided they reached the same final dose. Our analysis also did not demonstrate any clear differences in the incidence of adverse events across dosing regimens. Moreover, future studies should consider stratifying patients using validated tools such as the Leeds Assessment of Neuropathic Symptoms and Signs (LANSS) or the DN4 questionnaire to further clarify pregabalin’s role in individuals with prominent neuropathic features. For example, the study by Romano et al. found that pregabalin was particularly effective in patients with LANSS scores indicative of neuropathic pain, even when nociceptive components were also present (Romanò et al., 2009). The concept that pain with different mechanistic characteristics—nociceptive, neuropathic, or nociplastic—can coexist in the same patient, in any combination, has been further developed by Freynhagen et al. (2019) in their narrative review describing the phenomenon of ‘mixed pain.’ Given pregabalin’s demonstrated efficacy in both neuropathic and nociplastic pain models, it may be particularly well-suited for the management of these complex, overlapping pain phenotypes.

Pregabalin has demonstrated efficacy in spinal conditions beyond those included in the present meta-analysis. Specifically, pregabalin has been shown to be more effective than gabapentin in reducing pain associated with Failed Back Surgery Syndrome (Al-Ameri et al., 2024). Additionally, pregabalin has been associated with reductions in postoperative pain and opioid consumption following spinal fusion, highlighting its potential as an adjunct in postoperative pain management (Fujita et al., 2016). In line with these findings, the present meta-analysis revealed that pregabalin was associated with lower total costs compared to other interventions. This cost-effectiveness is further supported by evidence indicating that pregabalin’s efficacy in managing not only pain but also comorbid symptoms, such as anxiety and depression, contributes to a reduction in indirect costs, including loss of productivity and diminished quality of life, which are significant drivers of the economic burden in neurological disorders (Morera-Domínguez et al., 2010; Dagenais et al., 2008). These indirect costs are recognized as the most substantial component in the overall treatment burden for these conditions (Chodavadia et al., 2023).

Few meta-analyses have specifically examined the isolated effect of pregabalin for low back pain, which limits the ability to consistently analyze heterogeneity focused on pregabalin. For example, Wewege et al. (2023) reported only limited pain improvement with various medications, including pregabalin, and highlighted the low certainty of evidence for any pain reduction achieved with these agents. Similarly, Giménez-Campos et al. included pregabalin alongside gabapentin in their analysis, but only three studies specifically evaluated pregabalin. They found no evidence of efficacy for either drug in sciatica, with no benefit over placebo for pain relief and a worse safety profile, characterized by increased rates of dizziness and nausea/vomiting (Giménez-Campos et al., 2022). Consistent with these findings, Mascarenhas et al. (2023) observed that opioids provided greater pain relief than pregabalin in older adults with chronic nonspecific low back pain. Additionally, Shanthanna et al. (2017) found no benefit of pregabalin, even when used as an adjunct to opioids.

### 4.1 Limitations

A primary limitation of this meta-analysis is the inadequate reporting of pain etiology and symptom duration across the included studies, factors that are likely to influence pregabalin’s efficacy and contribute to the inconsistent outcomes observed, particularly in chronic cases (Romanò et al., 2009; Pota et al., 2012; Kalita et al., 2014; Park et al., 2024). Specifically, Kalita et al. (2014) included a mixed population of patients with neuropathic and non-neuropathic pain. The same situation was found in the case of Romanò et al. (2009) where the efficacy results of pregabalin were not stratified by neuropathic vs. non-neuropathic, presenting only aggregated results for the entire population. Additionally, Pota et al. (2012) mentioned that approximately 85% of the patients showed signs of a neuropathic pain component, they did not specify the criteria used for this determination or provide details about the specific underlying neuropathic conditions. In this sense, inadequate reporting of pain etiology is a critical limitation (these trials enrolled mixed populations or did not specify pain etiology in sufficient detail, which posed a significant challenge to conduct subgroup analyses stratified by pain type). Therefore it is essential to advocate for future research that more specifically addresses the underlying causes of pain, allowing for more precise analyzes based on etiology. Given this limitation, our findings should be interpreted with caution, especially when considering their applicability to mechanical or non-neuropathic LBP. There was also considerable heterogeneity in the types of pain assessed, with studies variably focusing on acute or chronic pain and on lumbar pain with or without radiculopathy, which complicates direct comparisons and affects generalizability. Another limitation of this review was the deviation from the originally registered PROSPERO protocol, which included EMBASE as a planned data source. Due to procedural adjustments, this search was not conducted during the initial search phase. However, a subsequent *post hoc* search in EMBASE using our predefined terms did not yield any additional unique articles. Moreover, we performed manual searching (handsearching) of reference lists from relevant studies to further minimize the risk of missing eligible publications, enhancing the thoroughness of our study selection process. A further limitation of this meta-analysis concerns the estimation of standard deviations, which in some cases had to be calculated using the Cochrane Review Manager software due to incomplete data reporting. The representativeness of certain subgroups was limited, as few studies specifically targeted these populations, and the wide variation and length of follow-up periods made it challenging to perform accurate subgroup analyses. Reporting of adverse events was inconsistent, with considerable variability in the timeframes for recording such events across studies, and the limited number of articles addressing each safety variable further compounded this issue. The potential for publication bias also exists and may have influenced the overall results. Also, there was a lack of evaluation of different pregabalin dosages, underscoring the need for future studies to determine the optimal therapeutic dose. Furthermore, significant differences among control groups—especially when pregabalin was compared with interventions other than placebo—and the scarcity of studies with a pregabalin-only control group limit the ability to draw definitive conclusions about its isolated effect. It should also be noted that the potential use of additional conservative treatments, such as electrotherapy, physiotherapy, rehabilitation, or exercise programs, could not be controlled for due to insufficient reporting in the included studies.

## 5 Conclusion

Pregabalin, whether administered as monotherapy or in combination with other treatments, has demonstrated efficacy in the management of LBP. Notable benefits include significant reductions in pain (observed from weeks four to eight of follow-up) as well as improvements in associated symptoms such as anxiety, depression, sleep disturbances, and overall quality of life. Additionally, pregabalin exhibited a safety profile comparable to that reported in existing literature and similar to other therapeutic alternatives, based on the adverse events analyzed. However, these findings should be interpreted with caution due to the overall limited and heterogeneous quality of the available evidence, particularly the inadequate reporting of pain etiology and symptom duration across studies. Several included trials enrolled mixed populations or did not clearly distinguish between neuropathic and non-neuropathic LBP, which limits the generalizability of the results and precludes robust subgroup analyses. Therefore, further well-designed, standardized studies are warranted to clarify pregabalin’s role across various LBP subtypes and to establish optimal dosing strategies.

## Supplementary material

SUPPLEMENTARY FILE S1 PubMed search strategy.

SUPPLEMENTARY TABLE S1 Assessment of the quality of studies through Methodological Index for Non-Randomized Studies (MINORS).

SUPPLEMENTARY TABLE S2 Treatment schemes and free-drug period of the included studies.
