# Supplementary material for: Efficacy and safety of pregabalin in the management of low back pain: a comprehensive meta-analysis
Source: Front Pharmacol. 2025 Sep 8;16:1659531. doi: 10.3389/fphar.2025.1659531 (PMC12451326; doi:10.3389/fphar.2025.1659531)

## 2.1 Supplementary table S1

Supplementary Table S1. Assessment of the quality of studies through Methodological Index for Non-Randomized Studies (MINORS).

| Study | Clearly stated aim | Consecutive patients | Prospective collection data | Endpoints | Assessment endpoint | Follow-up period | Loss less than 5% | Study size | Adequate control group | Contemporary group | Baseline control | Statistical analyses | MINORS |
| --- | --- | --- | --- | --- | --- | --- | --- | --- | --- | --- | --- | --- | --- |
| Gammoth et al. 2021 | 2 | 0 | 2 | 2 | 2 | 2 | 0 | 1 | 2 | 1 | 2 | 2 | 18 |
| Morera-Domínguez et al. 2010 | 2 | 2 | 2 | 1 | 1 | 2 | 0 | 2 | 1 | 2 | 2 | 2 | 19 |
| Saldaña et al. 2010 | 2 | 1 | 2 | 2 | 2 | 2 | 0 | 2 | 1 | 2 | 2 | 2 | 20 |
| Sicras-Mainar et al. 2013 | 2 | 0 | 0 | 1 | 2 | 2 | 0 | 2 | 2 | 2 | 2 | 2 | 17 |
| Taguchi et al. 2015: | 2 | 0 | 2 | 2 | 2 | 2 | 2 | 2 | 1 | 2 | 2 | 2 | 21 |


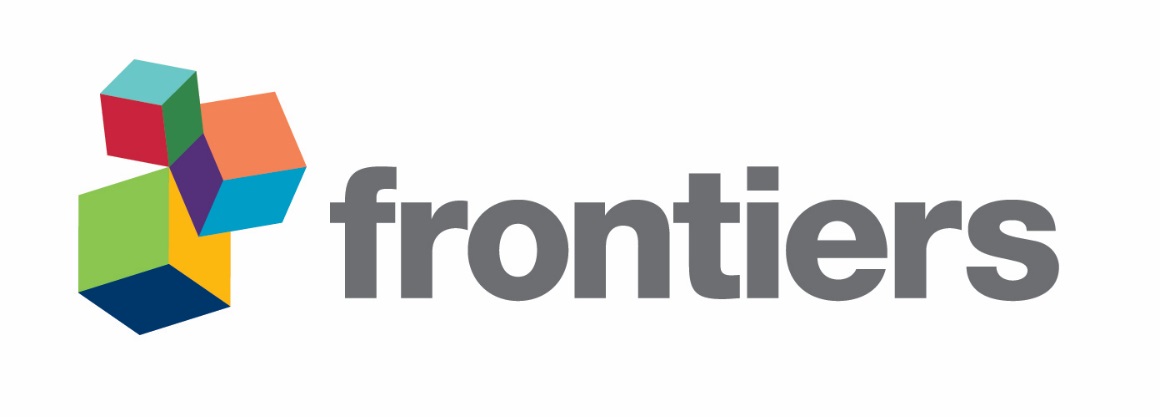

Supplement: Supplementary file 1 [file Table1.docx]
