# Supplementary material for: Efficacy and safety of pregabalin in the management of low back pain: a comprehensive meta-analysis
Source: Front Pharmacol. 2025 Sep 8;16:1659531. doi: 10.3389/fphar.2025.1659531 (PMC12451326; doi:10.3389/fphar.2025.1659531)

## 2.2 Supplementary table S2

Supplementary Table S2. Treatment schemes and free-drug period of the included studies.

| Study | Treatment schemes | Mean doses |
| --- | --- | --- |
| baron et al. 2010 | week single-blind pregabalin treatment phase using flexible-dose pregabalin 150–600 mg/day to identify responders (P30% pain reduction with pregabalin) who continued to the double-blind phase; 5-week double-blind treatment phase where patients were randomized to pregabalin (at the optimal | Mean and median were 445.9 and 600.0 mg |
| Baron et al. 2014 | After the randomization visit, patients were titrated to tapentadol PR 300 mg/day plus tapentadol PR 100 mg/day or tapentadol PR 300 mg/day plus pregabalin 150 mg/day. One week after the randomization visit, patients were further titrated to tapentadol PR 300 mg/day plus tapentadol PR 200 mg/day or tapentadol PR 300 mg/day plus pregabalin 300 mg/day. |  |
| chye et al. 2020 | Pregabalina a starting dose of 150 mg per day, adjusted to a maximum dose of 600 mg versus placebo | During 2 weeks…150-300; week 3-7 450mg to 600; week 8. 150-300 |
| Gammoth et al. 2021 | Pregabalin was initiated at 75 mg/day bedtime; thereafter, the dose was increased after four days to reach 300 mg/day in two divided doses. Gabapentin was initiated at 400mg/day at bedtime and increased to 800mg/day in two divided doses over five days | 4 days with 75mg/day |
| During almost 6 weeks 300mg/day |  |  |
| kalita et al. 2014 | Pregabalin was prescribed 75 mg twice daily for 2 weeks, followed by 150 mg twice daily for 4 weeks and then 300 mg twice daily. AMT was prescribed in a dose of 12.5 mg at bed time for 2 weeks followed by 25 mg for 4 weeks and then increased to 50 mg. | More than 300mg/day |
| kim et al. 2016 | All participants received treatments (limaprost, 5 μg 3 times per day; pregabalin, 75 mg 3 times per day; combination of limaprost and pregabalin, 3 times per day) on a 1:1:1 allocation basis for 8 weeks | 225 mg/day |
| Markman et al. 2015 | Pregabalin was started at 75 mg PO twice daily (active placebo, or diphenhydramine, 6.25 mg) and increased on day 4 to 150 mg PO twice daily (12.5 mg diphenhydramine) for 7 days. Pregabalin was decreased to 75 mg PO twice daily (6.25 mg diphenhydramine) on day 11 for 3 days of tapering (figure 1). If a subject could not tolerate 150 mg PO twice daily pregabalin (12.5 mg diphenhydramine), the subject was instructed to lower his or her dosage to 75 mg PO twice daily (6.25 mg diphenhydramine) for the remainder of the period, including the 3-day taper. | Between 150mg/dday and 300mg/day |
| mathieson et al. 2017 | The starting dose was 150 mg of pregabalin per day (75 mg twice daily) or matching placebo. The dose was adjusted to a maximum of 600 mg per day (300 mg twice daily), depending on the patient’s progress and the side effects at each dose level as assessed by the trial clinician. | During 2 weeks…150-300; week 3-7 450mg to 600; week 8. 150-300 |
| Morera-Domínguez et al. 2010 | Pregabalin (mean dose 189[141.7] mg/day), versus those who modified or added to their previous treatment an analgesic other than pregabalin | 189[141.7] mg/day |
| Park et al. 2024 | In the pregabalin group, 75 mg was administered twice daily (150 mg/day), while in the limaprost group, 5 mg was administered three times a day (15 mg/day). | 150mg/day |
| Pota et al. 2012 | Group A received transdermal buprenorphine 35 μg/h plus pregabalin 300 mg/day (two 150 mg tablets) for 3 weeks, with weekly control visits; Group B received transdermal buprenorphine 35 μg/h plus placebo (two tablets daily, com¬posed to look identical to pregabalin tablets) for 3 more weeks, with weekly control visits. | 300mg/day |
| Robertson et al. 2018 | First PGB 150mg to 600mg and GBP (400-800 mg) during 8 weeks, after it is changed. | From 150mg to 600mg/day |
| Romano et al. 2009 | During celecoxib + placebo treatment, mean celecoxib consumption was 4.12 ± 0.93 mg/kg/day; during pregabalin + placebo treatment it was 2.12 ± 0.69 mg/kg/day; and during celecoxib + pregabalin treatment it was 3.75 ± 0.86 and 1.78 ± 0.64 mg/kg/day, respectively. | Mean…<300mg/day |
| sakai et al. 2015 | 75-mg pregabalin before bedtime or tramadol/acetaminophen combination tablets (TRAM/APAP) twicedaily dosing (2 tablets per day; tramadol 75 mg and acetaminophen 650 mg per day) were administered randomly for 4 weeks | 150mg/day |
| Saldaña et al. 2010 | In the group receiving monotherapy with pregabalin, the mean dose was 187 ± 106 mg/day. The most frequently used drugs in the pregabalin add-on group (mean dose of pregabalin 191 ± 107 mg/day) were paracetamol (41.7%; mean dose: 2,146 ± 1,077 mg/day), tramadol (20.2%; mean dose: 164 1 81 mg/day), ibuprofen (20.5%; mean dose 1,218 ± 555 mg/day), and diclofenac (13.1%; mean dose 126 ± 52.3 mg/day). Non-pregabalin group took different drugs: the most frequent being paracetamol (37%, mean dose ± standard deviation: 2.144 ± 1.010 mg/day), metamizole (21%, 1,087 ± 455), tramadol (19%, 157 60 mg/day), ibuprofen (17%, 1.094 ± 451 mg/day), diclofenac (16%, 88 ± 49), gabapentin (13%, 989 ± 594 mg/day), tetrazepam (8%, 65 ± 24 mg/day), diazepam (6%, 6 ± 3 mg/day), fentanyl (6%, 59 ± 23 mg/day), codeine (6%, 59 ± 23 mg/day), and amitriptyline (5%, 38 ± 28 mg/day) | Monotherapy of pregabalin: 187 ± 106 mg/day/ pregabalin add-on 191 ± 107 mg/day |
| Sicras-Mainar et al. 2013 | Of patients receiving pregabalin (approved dose in the range 150– 600mg/day), 72.2% were taking doses <300 mg/day, while 68.4% of patients receiving gabapentin (approved dose 900–3600mg/day) were taking doses ≤900 mg/day. Only 9 patients (2.4%) received doses of pregabalin >600 mg/ day, and no patient on gabapentin received a daily dose >3.600 mg. The mean treatment duration was slightly lower with pregabalin than with gabapentin (5.2 vs. 5.4 months) | 72% <300mg/day |
| Taguchi et al. 2015 | Pregabalin dosing was flexible, and the range of doses among these patients was 25 mg/day to 300 mg/day versus usual care |  |
| Yeole et al. 2022 | pregabalin prolonged release (75 mg) and etoricoxib (60 mg) in comparison to etoricoxib (60 mg) alone during 8 weeks | 75mg/day |


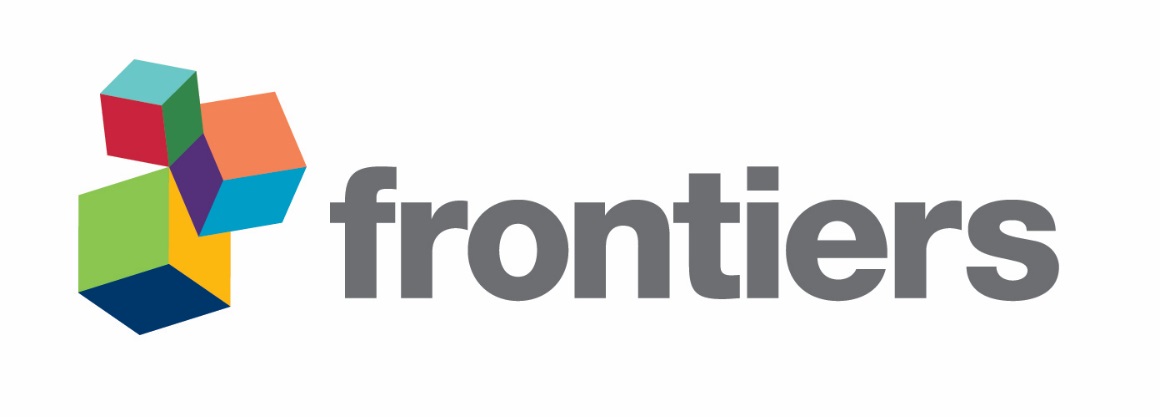

Supplement: Supplementary file 2 [file Table2.docx]
