# Supplementary material for: Efficacy and safety of pregabalin in the management of low back pain: a comprehensive meta-analysis
Source: Front Pharmacol. 2025 Sep 8;16:1659531. doi: 10.3389/fphar.2025.1659531 (PMC12451326; doi:10.3389/fphar.2025.1659531)

Supplementary Material

# `1. Supplementary Data

# 1.1 Supplementary File S1. Pubmed search strategy.

# Search: (Pregabalin OR lyrica) AND ("low backache" OR radiculopathy OR "spinal stenosis" OR "neurogenic claudication" OR sciatica OR "low back pain" OR lumbalgia OR lumbar OR spine)

# ("pregabalin"[MeSH Terms] OR "pregabalin"[All Fields] OR "pregabalin s"[All Fields] OR "pregabaline"[All Fields] OR ("pregabalin"[MeSH Terms] OR "pregabalin"[All Fields] OR "lyrica"[All Fields] OR "pregabalin s"[All Fields] OR "pregabaline"[All Fields])) AND ("low backache"[All Fields] OR ("radiculopathy"[MeSH Terms] OR "radiculopathy"[All Fields] OR "radiculopathies"[All Fields]) OR "spinal stenosis"[All Fields] OR "neurogenic claudication"[All Fields] OR ("sciatica"[MeSH Terms] OR "sciatica"[All Fields] OR "sciaticas"[All Fields]) OR "low back pain"[All Fields] OR ("low back pain"[MeSH Terms] OR ("low"[All Fields] AND "back"[All Fields] AND "pain"[All Fields]) OR "low back pain"[All Fields] OR "lumbalgia"[All Fields] OR "lumbalgias"[All Fields]) OR ("lumbarised"[All Fields] OR "lumbarization"[All Fields] OR "lumbarized"[All Fields] OR "lumbars"[All Fields] OR "lumbosacral region"[MeSH Terms] OR ("lumbosacral"[All Fields] AND "region"[All Fields]) OR "lumbosacral region"[All Fields] OR "lumbar"[All Fields]) OR ("spine"[MeSH Terms] OR "spine"[All Fields] OR "spines"[All Fields] OR "spine s"[All Fields]))

# Translations

# Pregabalin: "pregabalin"[MeSH Terms] OR "pregabalin"[All Fields] OR "pregabalin's"[All Fields] OR "pregabaline"[All Fields]

# lyrica: "pregabalin"[MeSH Terms] OR "pregabalin"[All Fields] OR "lyrica"[All Fields] OR "pregabalin's"[All Fields] OR "pregabaline"[All Fields]

# radiculopathy: "radiculopathy"[MeSH Terms] OR "radiculopathy"[All Fields] OR "radiculopathies"[All Fields]

# sciatica: "sciatica"[MeSH Terms] OR "sciatica"[All Fields] OR "sciaticas"[All Fields]

# lumbalgia: "low back pain"[MeSH Terms] OR ("low"[All Fields] AND "back"[All Fields] AND "pain"[All Fields]) OR "low back pain"[All Fields] OR "lumbalgia"[All Fields] OR "lumbalgias"[All Fields]

# lumbar: "lumbarised"[All Fields] OR "lumbarization"[All Fields] OR "lumbarized"[All Fields] OR "lumbars"[All Fields] OR "lumbosacral region"[MeSH Terms] OR ("lumbosacral"[All Fields] AND "region"[All Fields]) OR "lumbosacral region"[All Fields] OR "lumbar"[All Fields]

# spine: "spine"[MeSH Terms] OR "spine"[All Fields] OR "spines"[All Fields] OR "spine's"[All Fields]


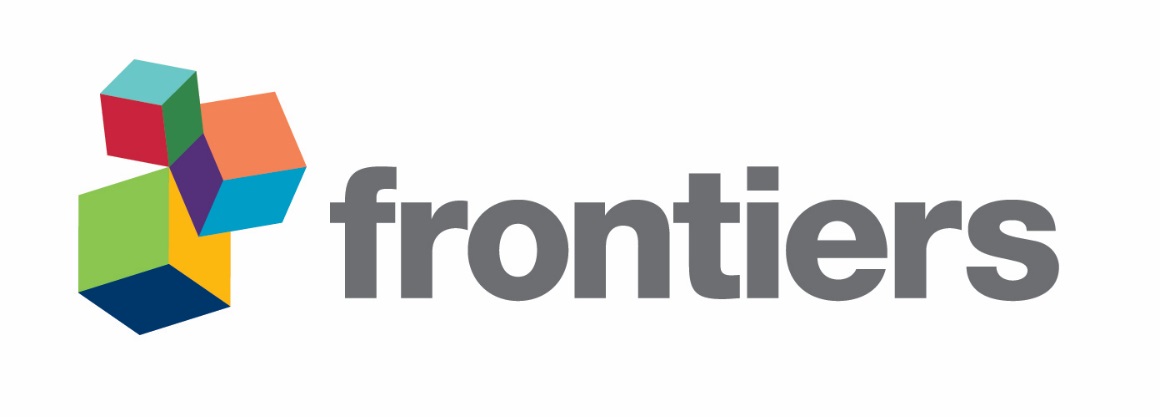

Supplement: Supplementary file 3 [file DataSheet1.docx]
